# The effects of a prior malignancy on the survival of patients with ovarian cancer: a population-based study

**DOI:** 10.7150/jca.46584

**Published:** 2020-08-25

**Authors:** Xiaoyuan Bian, Jiafeng Xia, Kaicen Wang, Qiangqiang Wang, Liya Yang, Wenrui Wu, Lanjuan Li

**Affiliations:** 1 State Key Laboratory for Diagnosis and Treatment of Infectious Disease, National Clinical Research Center for Infectious Diseases, The First Affiliated Hospital, Zhejiang University, Hangzhou, P. R. China.; 2 Collaborative Innovation Center for Diagnosis and Treatment of Infectious Diseases, Hangzhou, P. R. China

**Keywords:** Ovarian cancer, SEER, Prior malignancy, prognosis

## Abstract

**Background**: With the improvement in the prognostic outcomes of multiple malignancies, the population of cancer survivors is growing rapidly and is at higher risk of developing secondary ovarian cancer. However, the prevalence and clinical outcomes of prior cancer among newly diagnosed ovarian cancer patients remain unknown.

**Methods**: Patients diagnosed with ovarian cancer between 2004 and 2015 were identified using the Surveillance, Epidemiology, and End Results database. Patients were divided into two groups based on whether there was a prior malignancy. A multivariate Cox regression analysis was used to calculate all-cause and ovarian-specific survival. Furthermore, we conducted subgroup survival analyses of patients stratified by previous cancer site to explore the associations between prior cancer site and survival outcomes.

**Results**: A total of 52,182 patients with primary ovarian cancer were identified, and 3.6% (n=1,860) had a documented prior malignancy. In multivariate analyses, patients with prior malignancies had a worse all-cause and ovarian cancer-specific prognosis than those without. In subset analyses, patients with a history of thyroid cancer had a better all-cause and ovarian cancer-specific prognosis, and patients with prior colorectal, urinary system, skin, lung, haematologic and stomach cancers were at risk of decreased survival compared to that of patients without a prior cancer.

**Conclusions**: Prior malignancy has an adverse impact on the survival of patients with ovarian cancer, and the impact on prognostic outcomes varies by different prior cancer sites. The inconsistent survival effects of previous malignancies should be considered in clinical trial design and recruitment.

## Introduction

In the United States of America, ovarian cancer remains one of the most common tumours and the fifth most common cause of death in females according to 2020 cancer statistics 1. There have been many studies regarding the detection and therapy of ovarian cancer, but little is known about the clinical and prognostic factors of ovarian cancer as a second malignancy.

With the improvement in early diagnosis and better treatments, much progress has been made in the prognostic outcomes of multiple malignancies. The number of cancer survivors is increasing annually and is expected to reach approximately 20 million by 2026 2, 3. Studies have demonstrated that better survival expectations increase the possibility of developing a second primary malignancy among survivors of cancers 4-7, including ovarian cancer. Nearly 25% of elderly and 10% of younger cancer patients have had a pre-existing malignancy 8, and a higher risk of developing a second ovarian cancer has been observed in cancer survivors 9. Thus, there is a pressing need to understand the clinical characteristics and survival outcomes of these populations.

Recently, patients with prior malignancies were commonly excluded from most clinical trials due to the concern that a prior malignancy diagnosis would interfere with the implementation and efficiency of the treatment 10. A similar trend was observed in ovarian cancer trials in which a prior cancer was adopted as an exclusion criterion 11-13. However, the reasonableness of the exclusion criteria has barely been assessed, and evidence-based guidelines are urgently needed. Previous research has indicated that a history of malignancy does not result in an inferior prognosis of lung cancer, the effects vary by different prior cancer types and stages 10, 14, 15, and the validity of the criteria has not been evaluated in ovarian cancer patients. We therefore assessed the prevalence and clinical outcomes of prior cancer among newly diagnosed ovarian cancer patients by using the Surveillance, Epidemiology, and End Results (SEER) database.

## Methods

### Data Source

All data of patients with primary ovarian cancer were obtained from the SEER database, which is a population-based cancer database maintained by the National Cancer Institute. SEER*Stat (version 8.3.6) software was used to acquire the cohort based on SEER 18 Regs Custom Data (with additional treatment fields), Nov 2018 Sub (1975-2016 varying). This study was exempt from institutional review because the data were open-access and deidentified.

### Patients

In the present study, the cohort included women (≥18 years old) diagnosed with primary ovarian cancer (Site Recode ICD-O-3/WHO 2008 C56.9) between 2004 and 2015. Patients who were diagnosed based only on autopsy or death certificates or without information of active follow-up, survival months or previous malignancy status were excluded. In addition, patients with more than one prior cancer were also excluded. Records for prior malignancy, survival months, age at diagnosis, year of diagnosis, race, CA-125, histologic type, tumour size, AJCC stage (6th), tumour grade, insurance coverage, marital status, surgery, radiation and chemotherapy were obtained using the dataset.

Patient cases were divided into two groups based on whether there was a prior malignancy or not. We also excluded patients with a history of ovarian cancer to avoid the possibility of synchronous malignancy. In addition, we selected cases whose ovarian malignancy was diagnosed between 6 and 60 months following the diagnosis time of the first malignancy. *Six*-month exclusion was adopted to avoid the influence of synchronous primary cancers, and 60-month exclusion was a common exclusion criterion in clinical trials 10, 16. Next, patients with prior cancer were subdivided into groups according to the prior malignancy site as follows: breast, colon and rectum, uteri, urinary system, lung, skin, thyroid, haematologic, stomach, liver, other gastrointestinal and genitourinary, and other cancers.

### Statistical Analyses

Descriptive statistics were conducted to compare the baseline characteristics of the included ovarian patients with and without a previous malignancy. The distributions of prior tumour sites and mean interval times between previous cancer and ovarian cancer were calculated. Survival time was calculated from the time of ovarian cancer diagnosis to the last follow-up or death from any cause. Next, we adopted Kaplan-Meier survival curves of ovarian patients with and without prior cancer and conducted log-rank tests to estimate and compare the survival differences. In addition, we conducted nomograms to estimate the survival outcomes using R software. The C-index was used to measure the discrimination, and calibration plots were used to evaluate the predicted and actual probabilities. A multivariate Cox regression analysis of all-cause and ovarian-specific survival was conducted to estimate the prognostic effect of a prior malignancy after adjusting for variates, including age, race, CA-125, histologic type, tumour size, AJCC stage, tumour grade, insurance coverage, marital status and therapies. Furthermore, we conducted subgroup survival analyses of patients stratified by previous cancer site to explore the associations between the prior cancer site and survival outcomes. SPSS (version 20.0), GraphPad Prism (version 7.0) and R software (version 2.15.3) were used to conduct statistical analyses and images. A two-tailed P value of less than 0.05 was considered significant.

## Results

In the present study, we identified 52,182 patients with primary ovarian cancer diagnosed between 2004 and 2015. Among this cohort of patients, 3.6% (n=1,860) had a documented prior malignancy. In contrast to the cases without previous malignancies, patients with prior cancer were older (>65 years, 52.5% vs. 39.7%, P<0.001), were white (84.1% vs. 82.0%, P=0.002), had a smaller tumour size (24.5% vs. 19.7%, P<0.001), had fewer epithelial histologic types (64.9% vs. 67.1%, P<0.001), were less likely to show elevated CA-125 levels (57% vs. 66.4%, P<0.001) and enjoyed more insurance protection (71.4% vs. 71.3%, P=0.002). Regarding the therapeutic options, patients with prior cancer received less surgery therapy and chemotherapy (75.2% vs. 79.5%, P<0.001; 61.0% vs. 67.7%, P<0.001), and the administration of radiation therapy was similar between patients with and without a prior malignancy (1.3% vs. 1.0%, P=0.129). Additional clinicopathologic characteristics of the included patients are summarized in Table 1.

**Figure 1:**
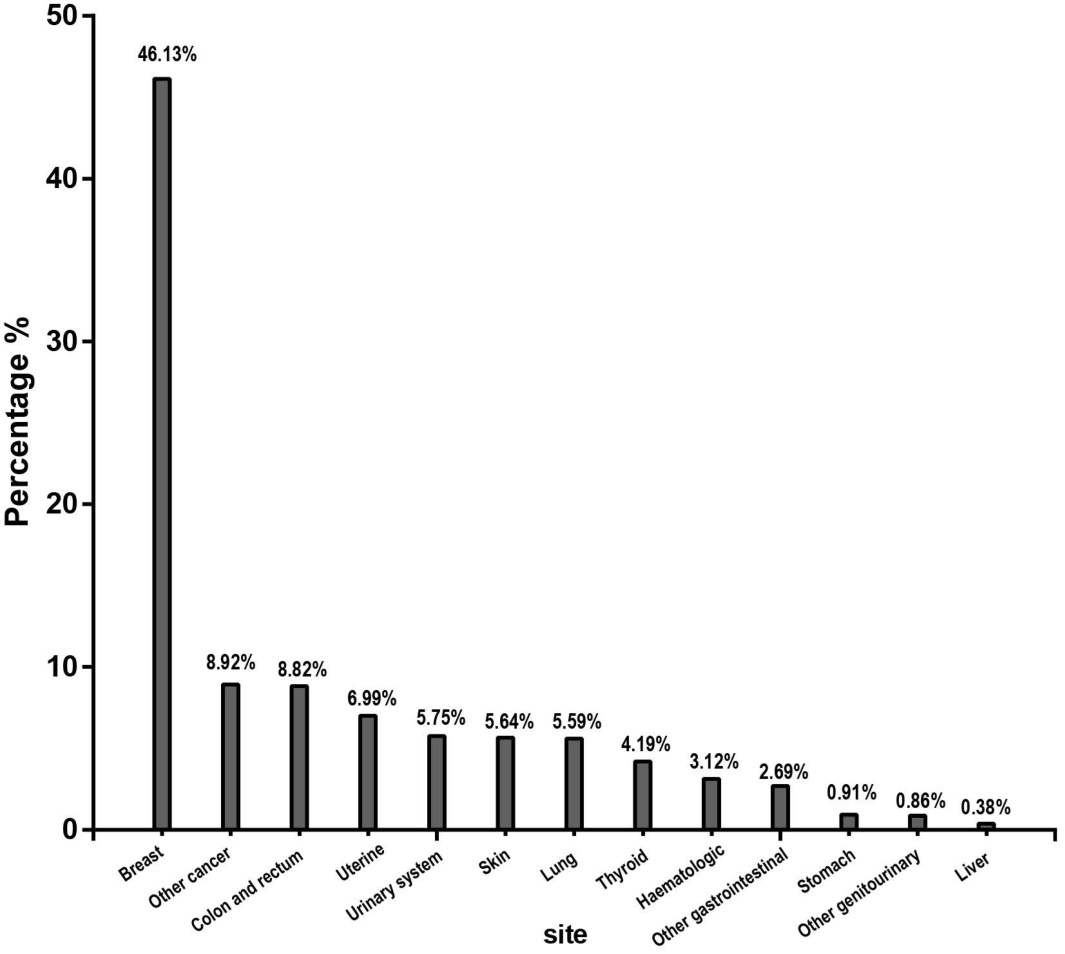
Type distribution of the most common prior cancer for patients diagnosed with ovarian cancer.

**Figure 2:**
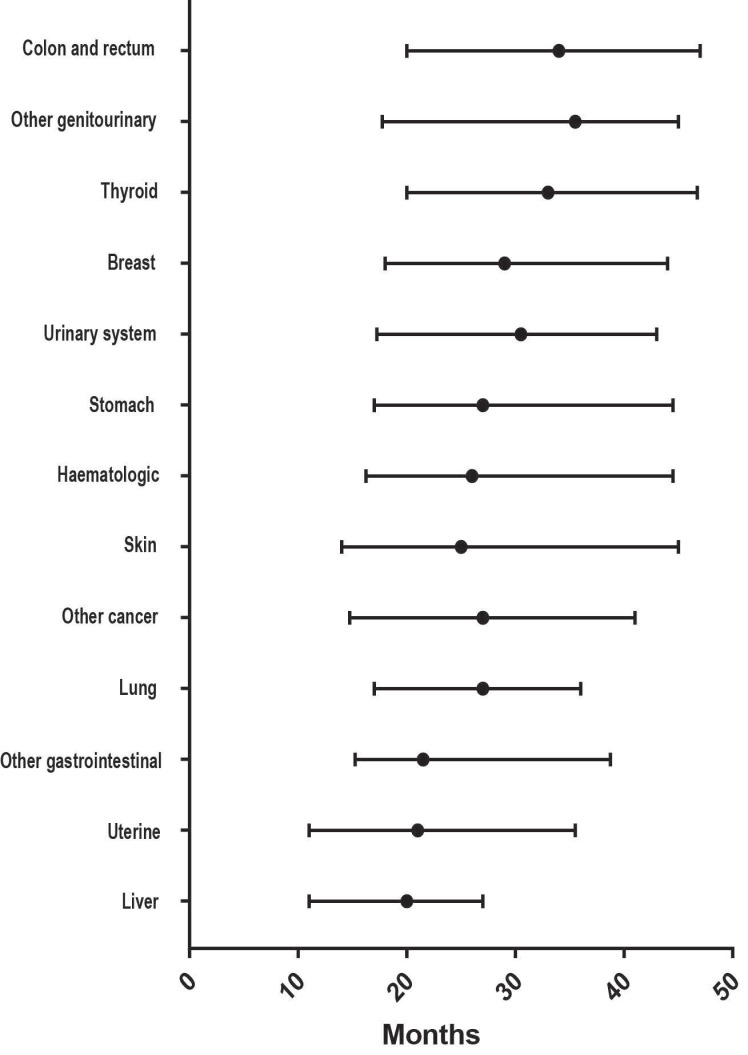
Distribution of the median interval time from prior cancer diagnosis to the subsequent ovarian cancer diagnosis.

Among the 1,860 individuals with a previous malignancy, the breast (46.13%) was the most common prior cancer site, followed by the colon and rectum (8.92%), uterine (8.82%), urinary system (5.75%), skin (5.64%), lung (5.59%), thyroid (4.19%), haematologic system (3.12%), stomach (0.91%) and liver (0.38%) (Figure 1). As shown in Figure 2, the median interval from the first malignancy to the subsequent ovarian cancer was 29 months, ranging from 20 months (liver) to 34.5 months (colon and rectum).

**Figure 3:**
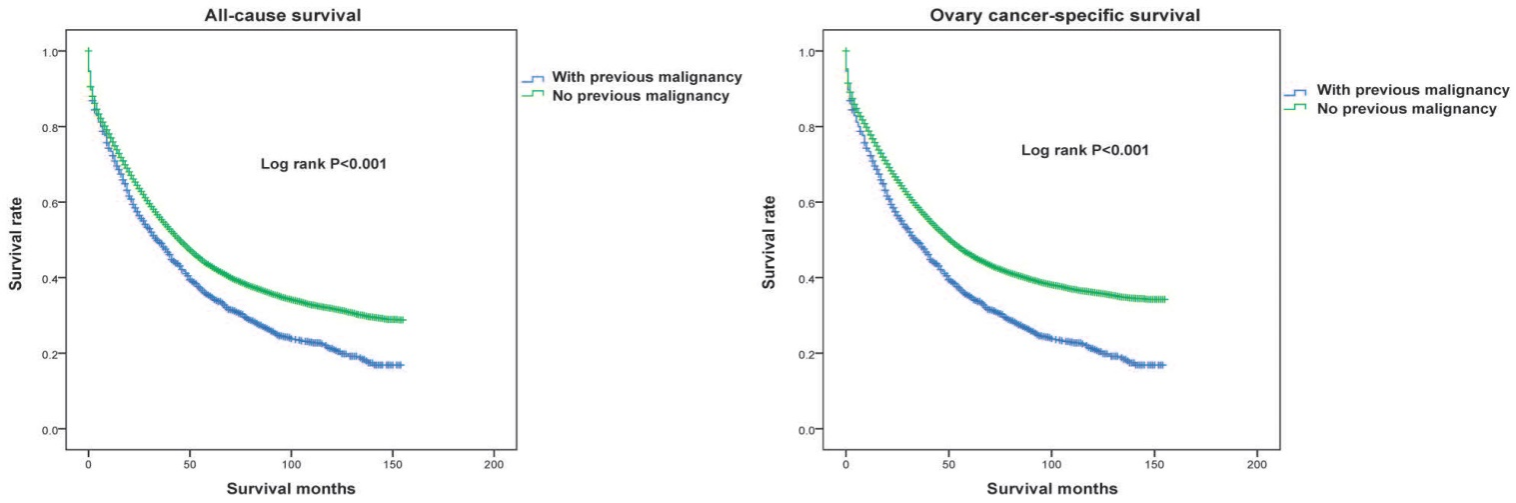
All-caused (left panel) and ovarian cancer-specific (right panel) survival between ovarian cancer patients with and without a prior cancer. All statistical tests were two-sided and P values were estimated and compared based on the log-rank test.

**Figure 4:**
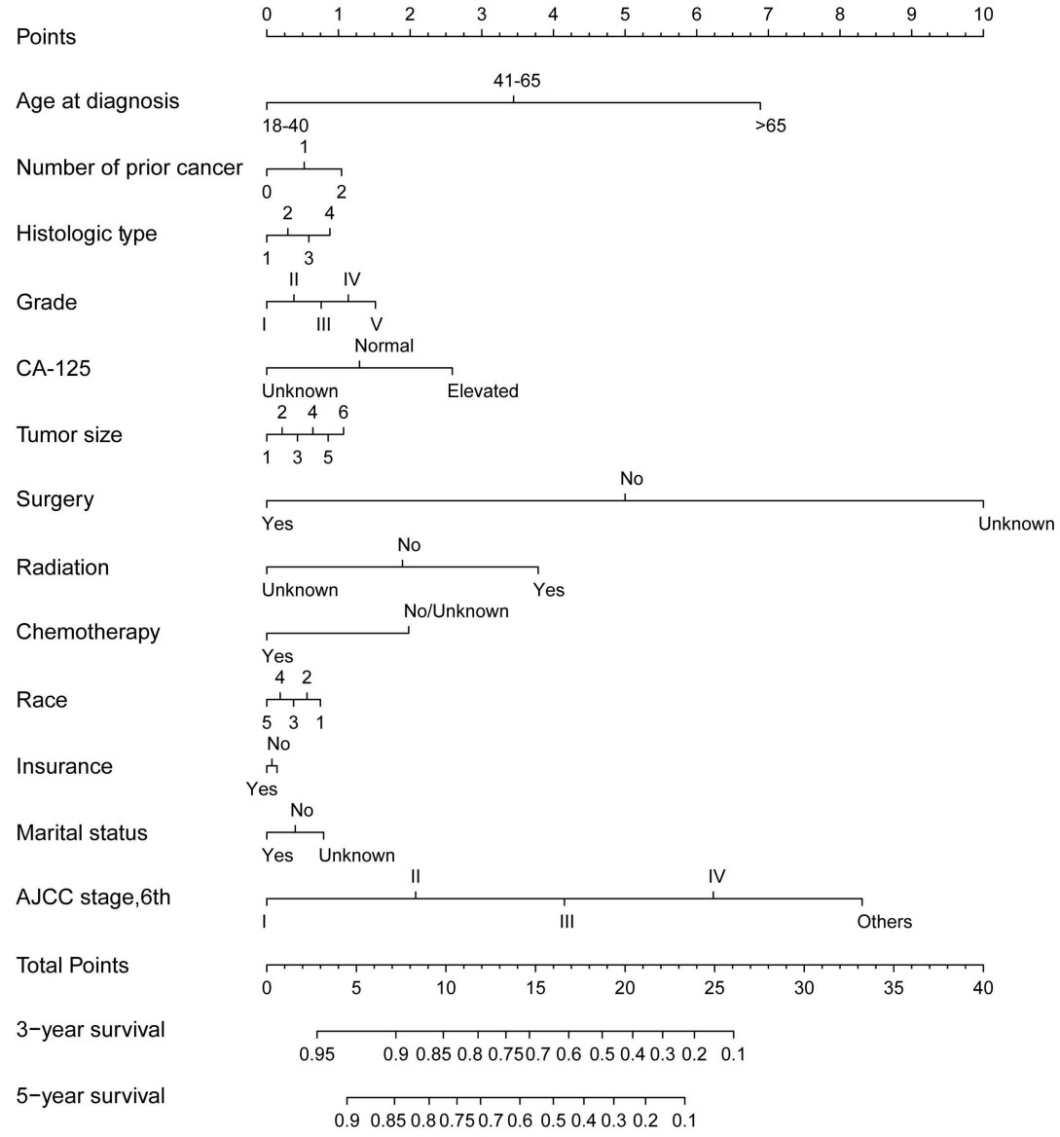
The nomogram for predicting all-caused survival in ovarian cancer patients with and without a prior cancer. Abbreviations: AJCC, American Joint Committee on Cancer.

**Figure 5:**
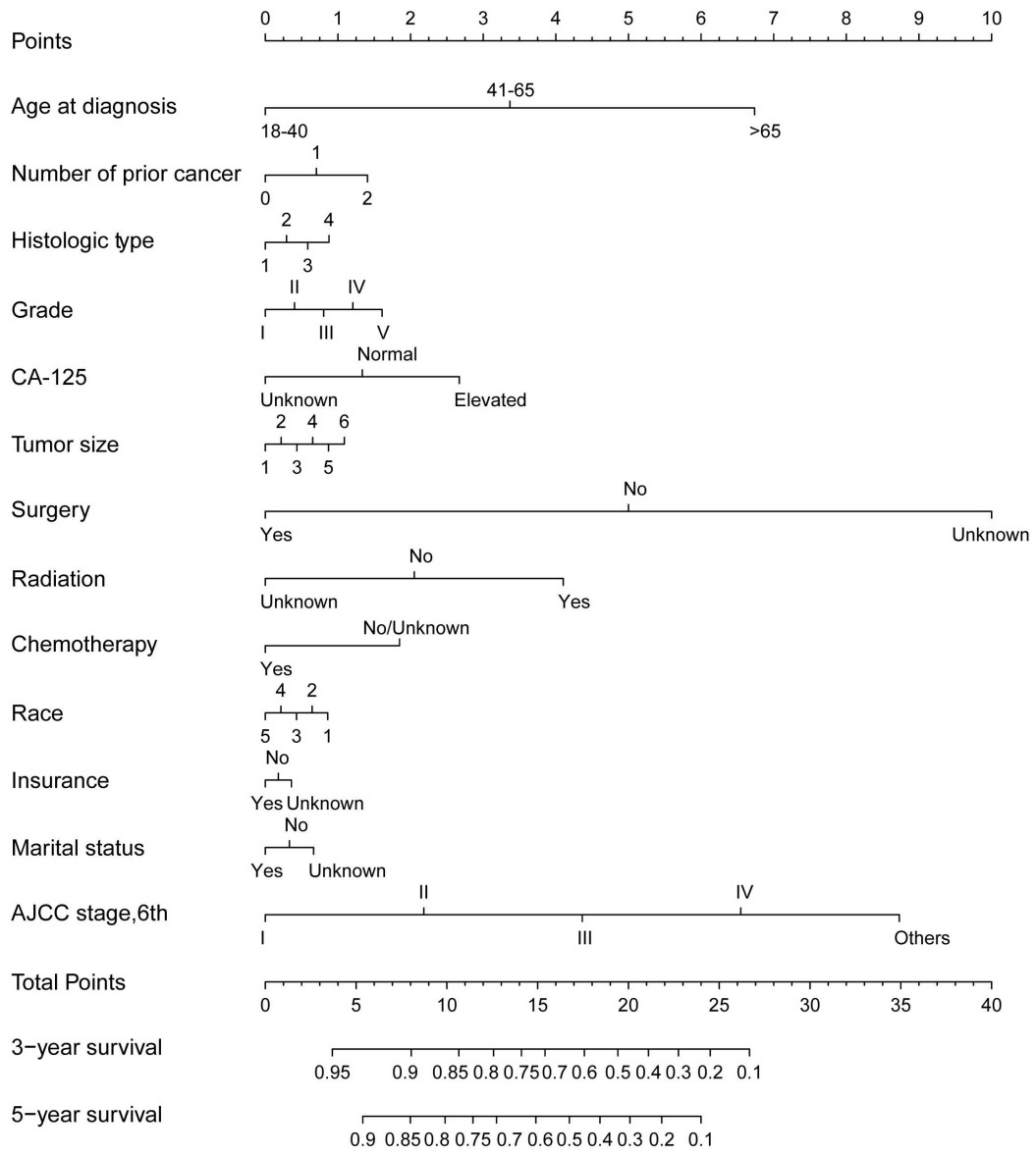
The nomogram for predicting ovarian cancer-specific survival in ovarian cancer patients with and without a prior cancer. Abbreviations: AJCC, American Joint Committee on Cancer.

Next, we conducted a Kaplan-Meier analysis to investigate the impact of prior malignancy on prognosis in patients with ovarian cancer. Significant differences were observed in both all-cause and ovarian cancer-specific survival rates between patients with and without prior malignancy (Both log rank P<0.001, Figure 3). The 3-year and 5-year all-cause survival rates of ovarian patients with prior malignancy were 49.0% and 35.1%, respectively, and those without prior malignancy were 55.4% and 43.2%, respectively (Table 2). When stratified by previous malignancy sites, patients with a history of breast, uteri and thyroid cancer had a better survival rate than those without prior malignancy, while patients with other types of prior malignancy had a worse survival (Table 2). We further adopted a prognostic nomogram to integrate all independent factors for all-cause and ovarian cancer-specific survival (Figures 4 and 5). The C-index of all-cause and ovarian cancer-specific prediction was 0.771 and 0.776, respectively (data not shown). The calibration plots of 3-year and 5-year survival are displayed in Figure S1 and S2, which showed optimal concordances between the predicted observations and actual outcomes.

The multivariate Cox regression analysis of the included patients is displayed in Table 3. Patients with a prior malignancy were associated with a worse all-cause and ovarian cancer-specific prognosis by contrast to those without, after adjusting for other variables such as age, race, CA-125, histologic type, tumour size, AJCC stage, tumour grade, insurance coverage, marital status and therapy options (HR=0.92, 95% CI=0.87-0.97, P=0.003 and HR=0.83, 95% CI=0.78-0.88, P<0.001). When compared to the individuals without a prior malignancy, patients with a history of thyroid cancer appeared to have a better all-cause and ovarian cancer-specific prognosis (Table 4). In contrast, compared to patients without a prior malignancy, patients with prior colon and rectum, urinary system, skin, lung, haematologic and stomach cancers were at a risk of decreased survival (Table 4).

## Discussion

An increase in cancer survivors has recently been observed due to advances in diagnostic and treatment strategies. With prolonged survival, patients are more likely to be subsequently diagnosed with a second primary malignancy. Determining the clinical significance and survival outcomes of prior malignancies is of great importance, as these factors might affect clinical trial design, oncological practice and therapeutic decisions. However, few studies have focused on patients with ovarian cancer who have a prior history of cancer.

In our large population-based study, we evaluated the effect of previous malignancies on the clinical characteristics and prognosis of patients with ovarian cancer. In our cohort, approximately 3.6% of patients had a documented prior malignancy within 5 years of the initial ovarian cancer diagnosis. Consistent with the results of previous studies 8, 17, a higher proportion (52.5%) was observed with a prior malignancy among elderly individuals (>65 years), indicating that elderly patients should receive more attention. Breast, colon and rectum, and uterine cancers were the three most common types of prior malignancies in our study. Breast and uterine cancers are the most common malignancies among females 18. Studies have reported that women with breast cancer are prone to develop primary ovarian cancer 19, 20. Genetic susceptibility and similar exposure factors might be the cause of multiple malignancies in ovarian cancer patients 21, 22. Patients with breast cancer have achieved prolonged survival time due to early detection and improved comprehensive therapies and are more likely to develop a second malignancy with inherited conditions 23. In addition, less elevated CA-125 levels and smaller ovarian cancer tumour sizes were observed in cancer survivors. This information indicated that those cancer survivors were given more intensive surveillance; therefore, it is necessary to screen for ovarian cancer as a second malignancy among cancer survivors. Moreover, the median interval from the first malignancy to the subsequent ovarian cancer varies by the initial malignancy site. This finding might provide clues for screening different malignancies according to the variations in time intervals 24.

Among the female population, ovarian cancer is one of the most malignant tumours, and the 5-year survival rate for the general population with ovarian cancer is approximately 47% 1, 25, 26. In the present study, the 5-year all-cause survival rate of ovarian cancer patients with a prior malignancy was 35.1%, which was much lower than that in patients without prior malignancies (43.2%) and the general population. Importantly, the multivariate analysis indicated that ovarian cancer patients with a pre-existing malignancy were associated with a more inferior prognosis than those without. This trend might be because patients with prior cancer received less surgery and chemotherapy, and therapies such as surgery and chemotherapy were negatively linked to the survival outcomes in our study. Similar results have also been found in non-Hodgkin lymphoma, stage IV colorectal cancer, lung cancer and prostate cancer 27-30. Conversely, earlier studies observed opposite results: a history of neoplasm did not result in a worse prognosis 31-33. Different population sizes and inclusion criteria for study populations (e.g., sex, age, stage, interval time) might contribute to the discrepancy.

To further evaluate the heterogeneous impacts of previous malignancy, we adopted a subgroup survival analysis of ovarian cancer patients stratified by specific prior cancer sites. Lung cancer remains the primary cause of cancer mortality in females according to 2020 cancer statistics 1. We observed that prior lung cancer was associated with significantly inferior survival in the current study. Consistently, Zhou et al. reported that lung cancer had a detrimental impact on the survival of prostate cancer patients 34. We speculate that the poor prognosis may be the only reflection of the aggressive biology and high fatality rate of lung cancer 17. As the third most common cancer 1, colorectal cancer was found to adversely impact all-cause and ovarian cancer-specific survival in our cohort. A previous study showed that colorectal cancer had a poor survival outcome in patients diagnosed with a previous malignancy 35. Similarly, survival detriments were also observed in the groups with stomach, urinary system, skin and haematologic cancers.

In contrast, decreased all-cause mortalities were observed in patients with prior thyroid cancer, indicating that certain types of prior malignancy might exert better effects on survival outcomes in patients with ovarian cancer. Consistent with our investigation, previous studies have reported that ovarian cancer patients with prior uterine cancer have either a favourable prognosis or no difference in prognosis when compared to patients without 36-38. In general, patients with inert cancers, such as thyroid and uterine cancers, were under more frequent and intensive healthcare, had less exposure to risk factors and received more medical resources and insurance protection 17, 39. These factors might result in favourable prognostic outcomes for patients with an initial cancer. Additionally, patients with a pre-existing breast cancer faced a worse ovarian cancer-specific survival than did patients without a prior neoplasm. Interestingly, no significant difference was observed in the all-cause survival between the two groups. In another study of patients with ovarian cancer following breast cancer, researchers found that individuals with prior breast cancer survived longer than those with ovarian cancer alone 40. BRCA1/2 mutation is a marker of hereditary breast and ovarian cancers and is related to improved survival among patients 41-44. However, the effect of the mutant gene on prognostic outcomes remains unknown, as data from BRCA carriers were unavailable in the database. Moreover, a history of liver cancer did not affect the prognosis for either all-cause or ovarian cancer-specific survival, which might be due to the reduction in exposure to risk factors such as smoking and alcohol.

Our study suggested that the inconsistent survival effects of previous malignancies should be considered in clinical trial design and recruitment. In most cancer trials, patients with prior cancer diagnosed within a past 5-year interval are excluded from enrolment 15, 45, 46. The reasons for the exclusion include the effects on outcomes, unclear adverse effects, tolerance or reduced efficiency of experimental treatments 30. However, the number of cancer survivors with a second cancer is increasing, and excessively restrictive inclusion criteria would limit the generalizability of the trials 8. Importantly, some clinical trials of ovarian cancer exclude patients with any previous malignancy, while some of the trials did not 11-13. Our results indicated that the impacts on the prognostic outcomes of ovarian cancer vary by the different types of prior cancer. Therefore, refinement of the inclusion criteria for patients with prior cancer is of great clinical importance. For instance, prior cancer therapy, rather than a prior cancer diagnosis, can be considered a criterion of exclusion. Clinical management of ovarian cancer with previous malignancies should be adopted in a site-dependent manner. Moreover, factors that might affect treatment tolerance (organ function, genetics and patient status) should be taken into consideration in inclusion or exclusion criteria 39. These strategies have already been adopted in multiple lung cancer trials 10, 39.

There are some limitations in the current study. First, complete information about lifestyle, genetics and environmental factors was not provided in the SEER database. In addition, the database lacked detailed data on complications and treatment, which may affect the survival outcomes of patients. Furthermore, the number of some specific prior cancer sites was too small to accurately conduct a statistical analysis, so they were grouped into other categories. Since this was a retrospective study, an intrinsic selection bias exists regarding patients with advanced malignancy who may not survive long enough to develop ovarian cancer. Last, there was a surveillance bias in that patients with prior cancer tend to be given more intensive surveillance and comprehensive therapies.

## Conclusions

In conclusion, the clinical characteristics of ovarian cancer patients with prior cancer significantly differed from those without prior cancer. A prior cancer had an adverse impact on the survival of patients with ovarian cancer, and the impact on prognostic outcomes varied by the different prior cancer sites. Therefore, the inconsistent survival effects of previous malignancies should be considered in clinical trial design and recruitment.

## Supplementary Material

Supplementary figures.

## Figures and Tables

**Table 1:** Baseline characteristics of included patients with ovarian cancer (n=52,182)

Patient Characteristics	Total N=52,182	With previous malignancy N=1,860 (3.6%)		No previous malignancy N=50,322 (96.4%)		*p*
**Age at diagnosis**						*<0.001*
18-40		65	(3.5%)	4,298 (8.5%)		
41-65		818	(44.0%)	26,071 (51.8%)		
>65		977	(52.5%)	1,938 (39.7%)		
**Year of diagnosis**						*0.887*
2004-2009		912	(49.0%)	24,589	(48.9%)	
2010-2015		948	(51.0%)	25,733	(51.1%)	
**Race**						*0.002*
White		1,565	(84.1%)	41,268	(82.0%)	
Black		156	(8.4%)	4,260	(8.5%)	
AI/AN		15	(0.8%)	341	(0.7%)	
AP		124	(6.7%)	4,220	(8.4%)	
Unknown		0	(0.0%)	233	(0.5%)	
**CA-125**						*<0.001*
Elevated		1,061	(57.0%)	33,437	(66.4%)	
Normal		182	(9.8%)	4,183	(8.3%)	
Unknown		617	(33.2%)	12,702	(25.2%)	
**Histologic Type**						*<0.001*
Epithelial		1,207	(64.9%)	33,758	(67.1%)	
Sex-cord-stromal		25	(1.3%)	970	(1.9%)	
Germ-cell		4	(0.2%)	1,061	(2.1%)	
Others		624	(33.5%)	14,533	(28.9%)	
**Tumour Size**						*<0.001*
0-1 mm		77	(4.1%)	1,713	(3.4%)	
1-5 mm		254	(13.7%)	5,680	(11.3%)	
5-10 mm		382	(20.5%)	9,770	(19.4%)	
10-20 mm		367	(19.7%)	12,343	(24.5%)	
>20 mm		69	(3.7%)	3,051	(6.1%)	
Unknown		711	(38.2%)	17,765	(35.3%)	
**AJCC stage (6th)**						*<0.001*
I		373	(20.1%)	10,494	(20.9%)	
II		166	(8.9%)	3,575	(7.1%)	
III		475	(25.5%)	14,221	(28.3%)	
IV		476	(25.6%)	13,740	(27.3%)	
Others		370	(19.9%)	8,292	(16.5%)	
**Grade**						*0.310*
I		109	(5.9%)	3,236	(6.4%)	
II		193	(10.4%)	5,692	(11.3%)	
III		554	(29.8%)	15,435	(30.7%)	
IV		331	(16.7%)	8,112	(16.1%)	
V		693	(37.3%)	17,847	(35.5%)	
**Surgery**						*<0.001*
Yes		1,399	(75.2%)	40,023	(79.5%)	
No		452	(24.3%)	9,976	(19.8%)	
Unknown		9	(0.5%)	323	(0.6%)	
**Radiation**						*0.129*
Yes		25	(1.3%)	496	(1.0%)	
No		1,833	(98.5%)	49,805	(99.0%)	
Unknown		2	(0.1%)	21	(0.0%)	
**Chemotherapy**						*<0.001*
Yes		1,134	(61.0%)	34,070	(67.7%)	
No/Unknown		726	(39.0%)	16,252	(32.3%)	
**Insurance**						*0.002*
Yes		1,328	(71.4%)	35,881	(71.3%)	
No		32	(1.7%)	1,553	(3.1%)	
Unknown		500	(26.9%)	12,888	(25.6%)	
**Marital status**						*0.756*
Married		901	(48.4%)	24,767	(49.2%)	
Unmarried		874	(47.0%)	23,373	(46.4%)	
Unknown		85	(4.6%)	2,182	(4.3%)	

Abbreviation: AI/AN, American Indian/Alaska Native; AP, Asian or Pacific Islander; CA-125, Cancer Antigen 125; Grade: I, well differentiated; II, moderately differentiated; III, poorly differentiated; IV, undifferentiated; V, unknown.

**Table 2:** 3-year and 5-year all-cause survival rate of ovarian cancer patients stratified by previous cancer site.

Previous cancer site	All-cause survival rate	
	3-year (95%CI)	5-year (95%CI)
No previous malignancy	55.4% (55.2%, 55.6%)	43.2% (43.0%, 43.4%)
With previous malignancy	49.0% (47.8%, 50.2%)	35.1% (33.9%, 36.3%)
Breast cancer	57.0% (55.3%, 58.7%)	41.5% (39.7%, 43.3%)
Colon and rectum cancer	37.6% (33.7%, 41.5%)	21.1% (17.5%, 24.7%)
Uterine cancer	56.6% (52.2%, 61.0%)	48.9% (44.2%, 53.6%)
Urinary system cancer	43.4% (38.4%, 48.4%)	26.6% (21.8%, 31.4%)
Skin cancer	49.3% (44.2%, 54.4%)	32.1% (26.9%, 37.3%)
Lung cancer	30.0% (25.2%, 34.8%)	17.5% (13.2%, 21.8%)
Thyroid cancer	65.8% (60.2%, 71.4%)	58.3% (62.2%, 64.4%)
Haematologic cancer	18.7% (13.3%, 24.1%)	10.4% (6.1%, 14.7%)
Other cancer	39.4% (35.5%, 43.3%)	26.7% (22.9%, 30.5%)

**Table 3:** Multivariable Cox regression analysis of all-caused and ovarian cancer-specific survival in patients with ovarian cancer.

Characteristics	All-cause adjusted HR	P	OC-specific adjusted HR	P
**Previous malignancy**				
Yes	Reference		Reference	
No	0.92 (0.87, 0.97)	0.003	0.83 (0.78, 0.88)	<0.001
**Age at diagnosis**		<0.001		<0.001
18-40	Reference		Reference	
41-65	1.54 (1.44, 1.65)	<0.001	1.47 (1.37, 1.58)	<0.001
>65	1.15 (1.12, 1.19)	<0.001	2.13 (1.99, 2.29)	<0.001
**Race**		<0.001		<0.001
White	Reference		Reference	
Black	1.18 (1.14, 1.23)	<0.001	1.16 (1.11, 1.21)	<0.001
AI/AN	1.10 (0.96, 1.26)	0.177	1.08 (0.94, 1.25)	0.292
AP	0.93 (0.88, 0.97)	0.001	0.91 (0.86, 0.95)	<0.001
Unknown	0.25 (0.18, 0.35)	<0.001	0.24 (0.17, 0.35)	<0.001
**CA-125**		<0.001		<0.001
Elevated	Reference		Reference	
Normal	0.61 (0.58, 0.65)	<0.001	0.61 (0.57, 0.65)	<0.001
Unknown	0.82 (0.80, 0.84)	<0.001	0.81 (0.79, 0.84)	<0.001
**Histologic Type**		<0.001		<0.001
Epithelial	Reference		Reference	
Sex-cord-stromal	0.41 (0.35, 0.48)	<0.001	0.37 (0.31, 0.44)	<0.001
Germ-cell	0.32 (0.25, 0.40)	<0.001	0.28 (0.22, 0.37)	<0.001
Others	1.15 (1.12, 1.18)	<0.001	1.16 (1.12, 1.19)	<0.001
**Tumour Size**		<0.001		<0.001
0-1 mm	Reference		Reference	
1-5 mm	1.40 (1.29, 1.52)	<0.001	1.40 (1.29, 1.53)	<0.001
5-10 mm	1.33 (1.23, 1.44)	<0.001	1.32 (1.21, 1.43)	<0.001
10-20 mm	1.28 (1.19, 1.39)	<0.001	1.28 (1.17, 1.39)	<0.001
>20 mm	1.50 (1.36, 1.65)	<0.001	1.46 (1.32, 1.62)	<0.001
Unknown	1.43 (1.33, 1.54)	<0.001	1.43 (1.33, 1.32)	<0.001
**AJCC stage (6th)**		<0.001		<0.001
I	Reference		Reference	
II	2.10 (1.95, 2.26)	<0.001	2.46 (2.26, 2.67)	<0.001
III	4.32 (4.09, 4.57)	<0.001	5.40 (5.07, 5.76)	<0.001
IV	5.94 (5.62, 6.29)	<0.001	7.53 (7.06, 8.04)	<0.001
Others	4.46 (4.21, 4.21)	<0.001	5.54 (5.18, 5.91)	<0.001
**Grade**		<0.001		<0.001
I	Reference		Reference	
II	1.82 (1.66, 1.99)	<0.001	1.99 (1.80, 2.21)	<0.001
III	2.18 (2.00, 2.37)	<0.001	2.43 (2.21, 2.68)	<0.001
IV	2.15 (1.97, 2.35)	<0.001	2.40 (2.17, 2.65)	<0.001
V	1.95 (1.79, 2.12)	<0.001	2.17 (1.97, 2.39)	<0.001
**Surgery**		<0.001		<0.001
Yes	Reference		Reference	
No	2.91 (2.81, 3.02)	<0.001	2.92 (2.81, 3.03)	<0.001
Unknown	1.53 (1.35, 1.74)	<0.001	1.51 (1.32, 1.73)	<0.001
**Radiation**		<0.001		<0.001
Yes	Reference		Reference	
No	0.76 (0.68, 0.84)	<0.001	0.76 (0.68, 0.85)	<0.001
Unknown	0.50 (0.28, 0.89)	0.018	0.45 (0.24, 0.86)	0.014
**Chemotherapy**				
Yes	Reference		Reference	
No/Unknown	1.65 (1.61, 1.70)	<0.001	1.61 (1.56, 1.65)	<0.001
**Insurance**		<0.001		<0.001
Yes	Reference		Reference	
No	1.12 (1.04, 1.21)	0.002	1.11 (1.03, 1.20)	0.007
Unknown	1.06 (1.04, 1.09)	<0.001	1.07 (1.04, 1.10)	<0.001
**Marital status**		<0.001		<0.001
Married	Reference		Reference	
Unmarried	1.16 (1.14, 1.19)	<0.001	1.14 (1.11, 1.17)	<0.001
Unknown	0.92 (0.87, 0.98)	0.006	0.88 (0.82, 0.93)	<0.001

Abbreviation: OC, Ovarian cancer; AI/AN, American Indian/Alaska Native; AP, Asian or Pacific Islander; CA-125, Cancer Antigen 125; Grade: I, well differentiated; II, moderately differentiated; III, poorly differentiated; IV, undifferentiated; V, unknown.

**Table 4:** Multivariable Cox regression analysis of all-caused and ovarian cancer-specific survival in patients stratified by previous cancer site

Characteristics	All-cause adjusted HR	P	Ovarian cancer-specific adjusted HR	P
**Previous cancer site**		<0.001		<0.001
**None**	Reference		Reference	
**Breast**	1.07 (0.98, 1.16)	0.147	1.17 (1.08, 1.28)	<0.001
**Colon and rectum**	1.69 (1.42, 2.02)	<0.001	1.85 (1.55, 2.21)	<0.001
**Uterine**	0.94 (0.74, 1.18)	0.582	1.03 (0.82, 1.30)	0.794
**Urinary system**	1.55 (1.24, 1.95)	<0.001	1.70 (1.36, 2.13)	<0.001
**Skin**	1.28 (1.00, 1.62)	0.046	1.40 (1.10, 1.78)	0.006
**Lung**	1.97 (1.59, 2.44)	<0.001	2.16 (1.74, 2.68)	<0.001
**Thyroid**	0.70 (0.49, 0.98)	0.037	0.76 (0.54, 1.08)	0.122
**Haematologic**	2.52 (1.91, 3.32)	<0.001	2.75 (2.01, 3.62)	<0.001
**Other gastrointestinal**	1.69 (1.22, 2.35)	0.002	1.85 (1.34, 2.57)	<0.001
**Stomach**	2.35 (1.42, 3.90)	0.001	2.58 (1.56, 4.29)	<0.001
**Other genitourinary**	1.31 (0.73, 2.36)	0.372	1.45 (0.80, 2.62)	0.218
**Liver**	1.65 (0.74, 3.67)	0.22	1.80 (0.81, 4.01)	0.150
**Other cancer**	1.67 (1.40, 1.99)	<0.001	1.83 (1.53, 2.18)	<0.001

Abbreviation: OC, Ovarian cancer; AI/AN, American Indian/Alaska Native; AP, Asian or Pacific Islander; CA-125, Cancer Antigen 125; Grade: I, well differentiated; II, moderately differentiated; III, poorly differentiated; IV, undifferentiated; V, unknown.
