# Supplementary material for: The effects of a prior malignancy on the survival of patients with ovarian cancer: a population-based study
Source: J Cancer. 2020 Aug 25;11(21):6178–87. doi: 10.7150/jca.46584 (PMC7532502; doi:10.7150/jca.46584)
Supplement: Supplementary file 1 — Supplementary figures. [file jcav11p6178s1.pdf]

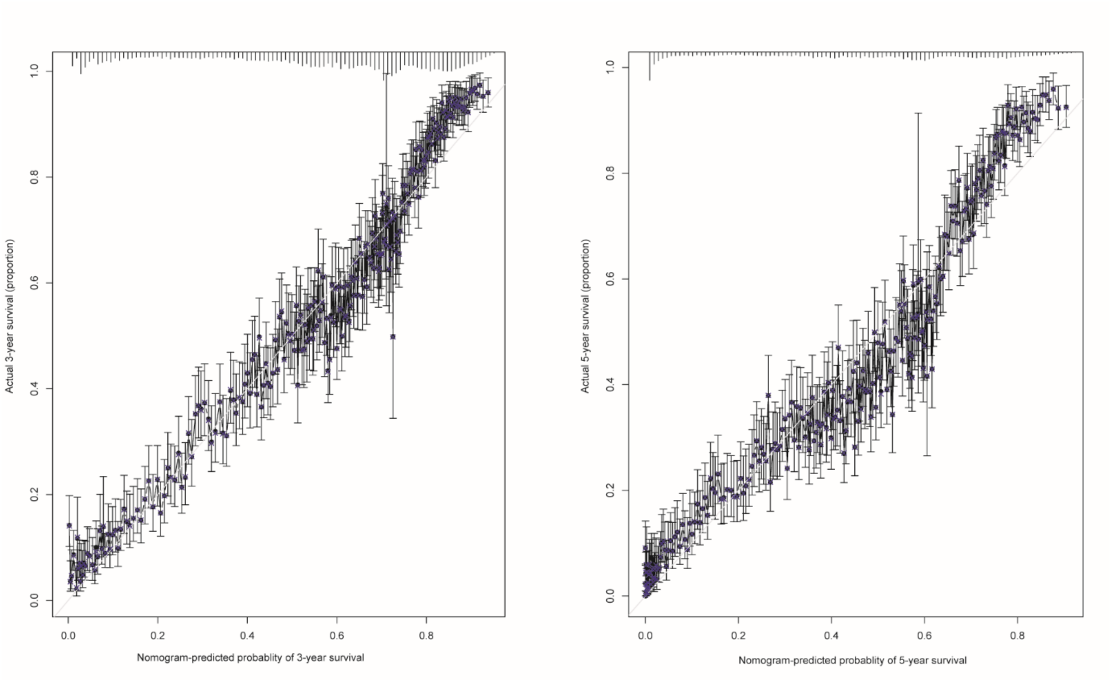

Figure S1 Calibration curve for predicting 3-year (left panel) and 5-year (right panel) all-caused survival in ovarian cancer patients with and without a prior cancer.

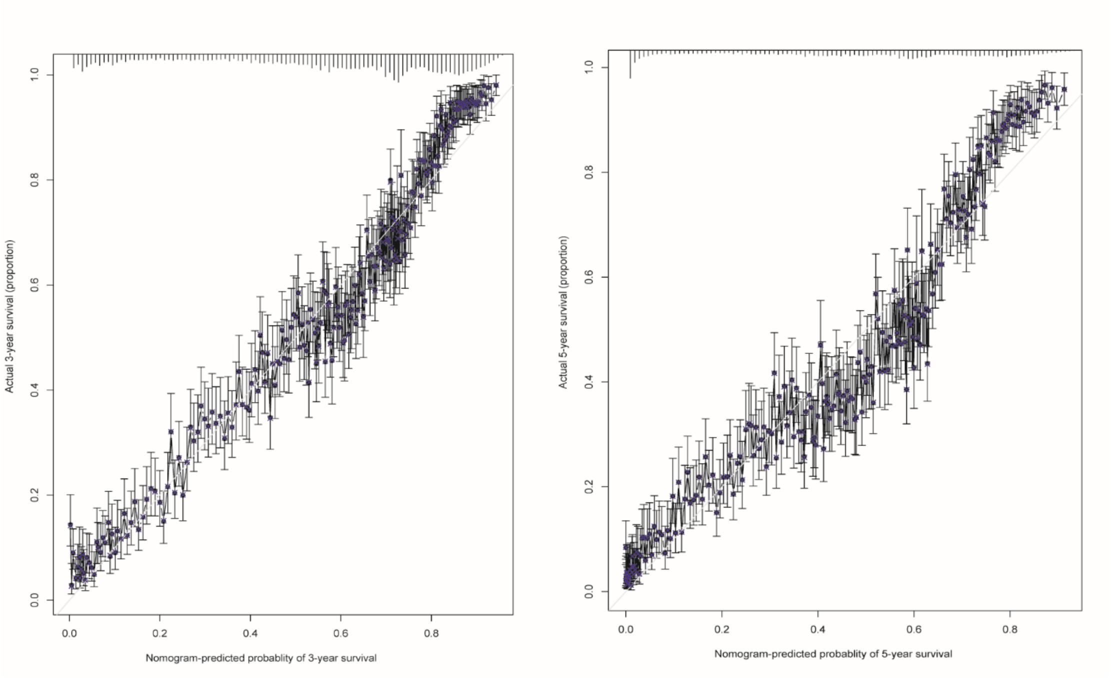

Figure S2 Calibration curve for predicting 3-year (left panel) and 5-year (right panel) ovarian cancer-specific survival in ovarian cancer patients with and without a prior cancer.
